# The Evolution of Blood Flow Restricted Exercise

**DOI:** 10.3389/fphys.2021.747759

**Published:** 2021-12-02

**Authors:** Eduardo D. S. Freitas, Murat Karabulut, Michael G. Bemben

**Affiliations:** ^1^Department of Health and Exercise Science, University of Oklahoma, Norman, OK, United States; ^2^Department of Health and Human Performance, University of Texas Rio Grande Valley, Brownsville, TX, United States

**Keywords:** kaatsu, occlusion training, practical BFR, resistance training, aerobic training

## Abstract

The use of blood flow restricted (BFR) exercise has become an accepted alternative approach to improve skeletal muscle mass and function and improve cardiovascular function in individuals that are not able to or do not wish to use traditional exercise protocols that rely on heavy loads and high training volumes. BFR exercise involves the reduction of blood flow to working skeletal muscle by applying a flexible cuff to the most proximal portions of a person’s arms or legs that results in decreased arterial flow to the exercising muscle and occluded venous return back to the central circulation. Safety concerns, especially related to the cardiovascular system, have not been consistently reported with a few exceptions; however, most researchers agree that BFR exercise can be a relatively safe technique for most people that are free from serious cardiovascular disease, as well as those with coronary artery disease, and also for people suffering from chronic conditions, such as multiple sclerosis, Parkinson’s, and osteoarthritis. Potential mechanisms to explain the benefits of BFR exercise are still mostly speculative and may require more invasive studies or the use of animal models to fully explore mechanisms of adaptation. The setting of absolute resistive pressures has evolved, from being based on an individual’s systolic blood pressure to a relative measure that is based on various percentages of the pressures needed to totally occlude blood flow in the exercising limb. However, since several other issues remain unresolved, such as the actual external loads used in combination with BFR, the type of cuff used to induce the blood flow restriction, and whether the restriction is continuous or intermittent, this paper will attempt to address these additional concerns.

## Introduction

Blood flow restricted exercise (BFR) has increasingly been used as an alternative training method for those unable to perform traditional aerobic or resistance exercise protocols, such as individuals with poor strength or endurance, recovering from an injury, or undergoing rehabilitation. BFR training may also serve as a supplemental training technique in combination with traditional exercise protocols to enhance adaptations to training based on the many published studies that demonstrate positive neuromuscular, aerobic, and anaerobic performance parameters (Abe et al., 2010a; Sudo et al., 2017; Amani-Shalamzari et al., 2019). These primary adaptations to BFR exercise include increases in muscle size and strength (Bjørnsen et al., 2019), improved physical function (Abe et al., 2010b), and enhanced endurance capabilities (muscular and cardiorespiratory; Held et al., 2020).

It is also worthwhile noting that the benefits associated with low-load BFR exercise are not limited to healthy young individuals (Abe et al., 2010a), but also middle-aged and elderly (Karabulut et al., 2010; Abe et al., 2010b; Ozaki et al., 2011b). Moreover, several studies have demonstrated the benefits of BFR training to clinical populations, such as post-surgery rehabilitating athletes (Kilgas et al., 2019) and individuals dealing with different forms of chronic inflammation and pain (i.e., arthritis, chronic fatigue syndrome, and fibromyalgia; McCully et al., 2004; Korakakis et al., 2018; Jørgensen et al., 2020). Thus, low-intensity aerobic exercise combined with BFR or low-load resistance exercise with BFR has emerged as potential training alternatives to traditional aerobic and resistance training programs, which are commonly performed at high intensities or using heavy loads, making it difficult for many of the populations already mentioned to withstand the training demands. However, the majority of published studies that explored BFR exercise have been acute interventions, primarily with college-aged males, necessitating the need for prolonged training studies and including females as subjects (Counts et al., 2018).

Although it is commonly accepted that BFR training is effective, there are several topics that need to be addressed to understand the evolution of this training method. First, BFR training has been found to be effective to improve several physiological systems, but improper use of the BFR technique can cause health problems; thus, it requires constant supervision, making its use outside of research and clinical settings difficult and challenging. Second, several studies were performed to understand the underlying mechanisms for BFR-related physiological adaptations, but many details still need further exploration and more invasive studies as well as potential animal models may be necessary to detail the main underlying mechanisms. Third, the devices used to restrict blood flow also varied between studies and have evolved over time. Instead of only the traditional electronically controlled pneumatic cuffs to induce restrictive pressures in a laboratory setting (KAATSU and KAATSU-mini, Sato Sports Plaza, Tokyo, JAP; Hokanson Rapid Cuff Inflation System, Bellevue, WA, United States; and the Delfi Medical Innovations Systems, Vancouver, BC, CAN), there is also a more practical BFR technique (Loenneke et al., 2010) that uses commercially available elastic wraps or bands to reduce blood flow during exercise. Even though having the ability to select different restriction cuffs may be useful, the differences in width and materials of the cuffs used to restrict blood flow can be problematic and result in inconsistencies in the findings. Finally, many previous studies have used different procedures to set restriction pressures and have used numerous different exercise training protocols.

Adding to the confusion, many details about how the cuff pressures were determined [initial pressure/tightness of cuff before inflation vs. final/target pressure – based on the original KAATSU philosophy (Sato, 2005)] are not clear or perhaps are not even reported. Fullana et al. (2005) reported that only a pressure greater than 40mmHg would change venous diameter at thigh level. In addition, several studies confirmed that the accumulation of some by-products, such as lactate and hydrogen ions, might cause increased afferent signals from intramuscular metaboreceptors resulting in an increased growth hormone response (Takarada et al., 2000a; Goto et al., 2005). Therefore, setting the initial tightness of the cuffs to 40mmHg or higher will apply some pressure on veins even when the cuffs are deflated, affecting the level of blood and by-product accumulation in the limbs, signals from muscle metaboreceptors, and venous return. A study by Karabulut et al. (2011b) reported supporting evidence of this theory that the session with greater initial tightness of the cuffs resulted in significantly lower tissue hemoglobin oxygen saturations and venous returns. Therefore, it is logical to assume that the lack of clear and descriptive information about BFR protocols has forced many researchers to develop their own specific BFR exercise protocols concerning the which muscle groups to study or what exercises should be used, what the appropriate number of sets or repetitions should be, the length of the rest periods between sets and exercise, etc. This has resulted in little to no consensus of what is the most effective BFR exercise protocol.

This review will describe the safety issues and concerns associated with BFR exercise, a summary of the potential mechanisms for positive adaptations, the types of restrictive devices available for exercise, how restriction protocols and exercise protocols have evolved since the first introduction of BFR exercise in the 1960s and 70s, aerobic and anaerobic exercise with BFR, and the limitations from previous studies and current gaps in the research literature.

## Safety Issues and Concerns

Perhaps the largest and most comprehensive study that examined the safety and efficacy of BFR exercise was completed in Japan in 2005 (Nakajima et al., 2006). Data were collected from 105 out of 195 facilities where BFR exercise was being used. Several types of facilities were surveyed: sports training gyms (27%), osteopathic facilities (24%), hospitals and clinics (22%), acupuncture facilities and other therapeutic massage facilities (10%), and rehabilitative centers (3%). The length of time that each facility had been using BRF exercise ranged from more than 5years (16%) to less than 1year (24%), with training sessions ranging from 5 to 30min, performed 1 to 3 times per week.

The most obvious concerns involve the cardiovascular system since the applied pressure directly affects blood flow return to the heart and central circulation and compressing blood vessels with the restrictive cuffs may also result in thrombosis and/or induce microvascular occlusion that could result in muscle cell damage and necrosis. Most often reported symptoms were dizziness and fainting due to the BFR-related reduced venous return and reduced cardiac preload. Surprisingly, the incidence of side effects reported in this large survey study was minimal and involved only a few incidences of venous thrombus (0.055%), pulmonary embolism (0.008%), and rhabdomyolysis (0.008%), resulting in the authors concluding that BRF exercise was a safe method of training for athletes, healthy individuals, and persons with various physical conditions (Nakajima et al., 2006).

In general, the potential effects of BFR exercise on the cardiovascular system depend on the level of restriction and the mode of exercise. Generally, the increases in heart rate, systolic blood pressure (SBP), and diastolic blood pressure are similar or perhaps even lower than high-load exercises performed with no restriction (Brandner et al., 2014; Mouser et al., 2018). Concerns regarding potentially dangerous spikes in blood pressure triggered by the exercise pressor reflex have also been raised (Spranger et al., 2015). Resistance exercise combined with BFR has been shown to induce robust metabolic alterations in terms of accumulation of several metabolic by-products, such as lactate, inorganic phosphate, dihydrogen phosphate, and hydrogen ions (Suga et al., 2009, 2010). Due to the occlusion of venous return to the central circulation, metabolites are trapped and accumulate locally in the active musculature. Thus, there is some concern regarding the possibility of the elevated exercise-induced metabolic response generating exaggerated increases in blood pressure *via* the exercise pressor reflex (Spranger et al., 2015). However, there is currently no data demonstrating that low-load resistance exercise with BFR elicits such rapid and exaggerated increases in blood pressure. In fact, Pinto et al. (2018) investigated the cardiovascular and metabolic response of hypertensive women to low-load BFR versus traditional high-load resistance exercise and demonstrated that BFR resistance exercise induced neither an exaggerated nor greater cardiovascular or metabolic response compared to traditional resistance exercise. Additionally, studies by Kambič et al. (2019, 2021) have reported that BFR training is safe and is associated with significant improvements in muscle strength and improved muscle function in patients with coronary artery disease (CAD) and that 8weeks of BFR resistance training can significantly lower systolic and lower diastolic blood pressure with no changes in N-terminal prohormone B-type natriuretic hormone, fibrinogen, and D-dimer values in patients with CAD. Large artery compliance increased the same amount and small artery compliance less than observed with high-load exercise without restriction (Fahs et al., 2012), but another study reported that small arteries stay stiff for a longer period of time following acute vibration exercises in combination with blood flow restriction (Karabulut et al., 2018). With respect to the possible development of a thrombosis (as indicated by an increase in D-dimer) with BFR exercise, no reports have been found in the existing literature (Fry et al., 2010). Also, C-reactive protein, which is linked to clot formation, has also shown to be stable following BFR exercise (Nakajima et al., 2010).

Other potential concerns associated with BFR exercise have included increases in metabolic activity and possible muscle damage. Current findings indicate that BFR exercise does not increase oxidative stress or antioxidant defense (Goldfarb et al., 2008; Petrick et al., 2019). Regarding muscle damage, most studies indicate that BFR exercise does not seem to result in significant muscle damage (Loenneke and Abe, 2012; Karabulut et al., 2013; Thiebaud et al., 2013; Alvarez et al., 2019), probably due to the fact that the low training loads commonly used (i.e., 20 to 50% of 1RM) are not high enough to induce substantial mechanical stress. However, the possibility of muscle damage and even rhabdomyolysis episodes in response to BFR exercise have not been completely been ruled and are still debated in the literature (Loenneke et al., 2014b; Burr et al., 2020; Wernbom et al., 2020). It should be noted that even though serious injuries are not common in the studies that use lab-based BFR units, which can set and control the pressure used during training, the use of elastic bands or wraps that cannot determine, set, or control the pressure can be problematic. Therefore, improper use of field-based, lab-based, or any other BFR techniques that cannot determine the pressure being used may result in serious health issues and can be unsafe if total occlusion occurs.

## Potential Mechanisms

Although the long-term benefits of BFR exercise, including increased muscular size, strength, muscular endurance, muscular power, and enhanced aerobic capacity, have been extensively reported in the scientific literature (Laurentino et al., 2012; Bjørnsen et al., 2019; Wilk et al., 2020), the underlying mechanisms responsible for these adaptations generally remain speculative. One of the most often referenced occurrence that is thought to be linked to the positive adaptations of BFR exercise is the exercise-induced metabolic stress (Suga et al., 2010; Takada et al., 2012). Although BFR exercise is commonly performed at low loads (20–50% of maximal strength), most studies report a pronounced metabolic response as confirmed by the accumulation of metabolic by-products, such as lactate, di-protonated phosphate, deoxygenated hemoglobin, inorganic phosphate, and hydrogen ions and often at similar levels as those caused by traditional high-load resistance exercise in many studies (Suga et al., 2009, 2012; Yasuda et al., 2010a; Karabulut et al., 2014).

This enhanced metabolic response to BFR exercise is important for several reasons. First, the accumulation of metabolic by-products thought to be associated with the activation of groups III and IV afferent nerve fibers that inhibit the α-motoneurons that innervate slow-twitch motor units, thus requiring the early recruitment of the fast-twitch motor units which are more responsive to hypertrophic adaptations. Additionally, the BFR exercise-induced metabolic response is also linked to the acute release of anabolic hormones like growth hormone (GH) which could then result in an increase in insulin-like growth factor-1 (IGF-1) and ultimately an increase in vascular endothelium growth factor to stimulate vasculogenesis to accommodate the need for a greater blood supply to the increases in muscle mass. In fact, Takarada et al. (2000a) reported an increase in blood lactate levels following a single bout of BFR exercise coupled to an approximately 290 times increase in GH compared to baseline levels.

The central chemoreceptors, located within the ventrolateral medulla, respond to alterations in [H+] and indirectly to CO_2_ and peripheral chemoreceptors, located within the carotid and aortic bodies, respond to changes in PaO_2_, PaCO_2_, and pH of the arterial blood. In addition, since baroreceptors sense the changes in blood pressure (Farell et al., 2011), variation in cuff pressure may affect the level of venous return. Therefore, it can be speculated that stimulation of the central and peripheral chemoreceptors by the accumulated metabolites after deflating the BFR cuffs and the reduced end diastolic volume (preload) due to the BFR cuffs-related reduced venous return may be responsible for the changes in several physiological systems and training-related adaptations.

A third potential underlying mechanism to explain the positive adaptations associated with BFR exercise is the muscle swelling that results from the induced exercise demands (Loenneke et al., 2012a). Takarada et al. (2000b) applied a BFR stimulus, without exercise, to patients following anterior cruciate ligament surgery. Each session consisted of 5 sets of BFR application, at pressures between 200 and 260mmHg, separated by 3-min rest intervals, for 10days following surgery. They reported a diminished disuse atrophy of the knee extensor muscles compared to a control group (9.4% vs. 20.7%, respectively). Since BFR was applied in the absence of exercise, the contributions of a localized metabolic response or increased muscle activity were unlikely contributors to the adaptations observed (Loenneke et al., 2012c) leading to the speculation that activation of localized chemoreceptors or exercise-induced muscle swelling, commonly observed following BFR exercise, may play a role in the exercise hypertrophic response by shifting the protein balance toward anabolism. This is still speculative since a recent study demonstrated that the application of BFR in the absence of exercise did not increase myofibrillar protein synthesis, except when combined with exercise (Nyakayiru et al., 2019).

A fourth potential mechanism to explain the adaptations associated with BFR exercise is an increased protein synthesis by altering biomolecular pathways, including the mammalian target of rapamycin complex 1 (mTORC1) and the inhibition of atrogenes like Muscle RING Finger1 (MuRF1) and atrogin-1 and the inhibition of the myostatin (MSTN) pathway. Fry et al. (2010) reported that a single bout of BFR exercise at 20% of 1-RM increased muscle protein synthesis by 56% in comparison with pre-exercise levels as well as increasing the phosphorylation of the mTORC1 downstream target ribosomal S6 kinase 1 (S6K1). BFR exercise has also been shown to influence the MSTN pathway, which is a down regulator of muscle growth. In this context, Laurentino et al. (2012) demonstrated that 8weeks of BFR resistance training resulted in similar muscular size and strength gains similar to traditional high-load resistance training with concomitant decrease in MSTN gene expression.

## Types of Restrictive Devices (Lab- and Field-Based)

The blood flow restriction devices commonly used are basically divided into two major groups: traditional (laboratory-based) and practical (field-based). Traditional laboratory-based BFR devices include the original KAATSU-Master and -Mini (Sato Sports Plaza, Tokyo, Japan), the Delfi Personalized Tourniquet System (Delfi Medical Innovations System, Vancouver, BC, CAN), and the Hokanson rapid cuff inflation system (Hokanson, Inc., Bellevue, WA, United States), with additional models being designed and released as BFR exercise training gains in popularity. KAATSU, Delfi, and Hokanson systems are electronically controlled units. The KAATSU, Hokanson, and Delfi units use standardized cuffs of varying widths and lengths which are physically attached to the devices with cords and provide the capability of precisely controlling the amount of pressure applied to each limb during exercise (McEwen et al., 2019). However, disparities in features between the units results in differences in how the restrictive pressures are set [based on limb occlusion pressure (LOP)]. A study by Weatherholt et al. (2019) compared the Delfi and KAATSU devices to investigate the effects of cuff width on LOP and reported significant differences between units for LOP (Delfi: 239.4mmHg vs. KAATSU: 500mmHg). Although these devices are precise and relatively easy to use, they are quite expensive, and somewhat confined to laboratory or clinical settings, making it difficult to perform BFR exercise in more practical settings, such as gyms.

The inability to use traditional BFR in applied settings, like gyms and practice facilities, has stimulated the development of more practical approaches to traditional BFR exercise. Practical BFR exercise consists of using elastic bands or wraps placed around the exercising limbs and not connected to any external pressure controlling device. These techniques are much more affordable and widely available for sale online and require little to no training but have the potential to be over-tightened by those not familiar with the theory of BFR exercise. Since this type of training does not allow for precise control of the pressure that is being applied during exercise, care should be used to ensure that the total occlusion of blood flow does not occur. Wilson et al. (2013) proposed using a perceived tightness scale in an attempt to prescribe the restrictive pressure, although the reliability of this method has been questioned (Bell et al., 2020). Recently, a more practical approach called “capillary refill time (CRT)” has also been used to set and adjust the restriction pressures. CRT is a measure of the time that takes for the capillary bed to regain its color after pressure has been applied. CRT is used in clinical settings to evaluate different populations such as children (Fleming et al., 2016) and the elderly (Schriger and Baraff, 1988) with various health problems like circulatory failure, hemorrhagic fever, and peripheral perfusion (Silverstein and Hopper, 2015; Gallier, 2020). If CRT takes more than a few seconds depending on the population and where it was measured (finger, hand, foot, leg, etc.), it is considered as a health problem or a sign of poor perfusion (Fleming et al., 2015; Silverstein and Hopper, 2015). During BFR exercise training, the CRT is determined by pressing the thumb into the quadriceps muscle immediately above the knee (for the leg pressure cuff) or into the palm of the hands (for the arm pressure cuff) and releasing to see how quickly (in seconds) the blanched (white) area returns to normal color. The pressure that allows the normal color to be regained within 2 to 3s is then used as the final/target pressure (Amano et al., 2016). However, it should be noted that a study reported that CRT was dependent on patient and environmental factors (Anderson et al., 2008), therefore, even though CRT has been used in clinical settings, using CRT for the purpose of BFR training is new and may need additional research to provide more details regarding this technique.

## The Evolution of Determining Restrictive Pressures and Exercise Protocols

The BFR technique originated in Japan by Yoshiaki Sato around the year 1960. The process began with self-experimentation, with Sato using the technique to rehabilitate from a fracture on his leg BFR exercise then gained public notoriety and made its way into research (Sato, 2005). The original research that used the KAATSU-Master device based restrictive pressures on a person’s upper body SBP (Wernbom et al., 2006) or simply used arbitrary occlusive pressures around 200mmHg (Takarada et al., 2000a). For arm BFR exercise, pressures that corresponded to 120% of upper body SBP were used (Arm SBP×1.2). For leg BFR exercise, an additional 20% restriction was added to the 120% of upper body SBP cuff pressure (Arm SBP×1.44; Sato et al., 2005; Takano et al., 2005). This essentially resulted in a constant, uniform restriction pressure for all individuals who had similar SBPs, regardless of age, condition, limb size, or limb composition. This approach is similar to suggesting that all individuals should exercise with the same weight or run at the same pace, regardless of an individual’s specific characteristics. The use of arbitrary fixed pressures is also currently contraindicated as these may correspond to pressures above the persons’ total occlusive pressure, thus increasing the risks of nerve and ischemic injuries (McEwen et al., 2019).

More recently, the basic concept of “individualization” has been used to set the restrictive pressures during traditional BFR exercise to various percentages of the total arterial occlusion pressure for an exercising limb as measured *via* Doppler ultrasound or predicted using standardized equations developed using biometrical measures, such as limb circumference (Laurentino et al., 2018). These pressures are usually in the range of 40 to 60% of the total occlusion pressure for the limb. Additionally, similar long-term neuromuscular adaptations have been reported with restrictive pressures ranging from 40 to 90% of total occlusion (Counts et al., 2016). As a safety concern, prior to formal inclusion into any BFR exercise study, the individual’s ankle-brachial index should be assessed to screen for peripheral artery disease and exclusion as a potential subject.

It should be noted that setting the restrictive pressure at 50% of the total occlusion pressure to a given limb does not mean that blood flow to the exercising limb has been reduced to exactly 50% of its normal flow at baseline. Mouser et al. (2017) assessed brachial blood flow in the arms of 45 men and women between the ages of 18 and 40years, using color flow mode and Doppler velocity waveforms at 10% of total occlusion pressure, 20% total occlusion pressure, and up to 90% total occlusion pressure, increasing each measurement by 10% intervals. Results indicated that blood flow decreased in a nonlinear, stepped fashion but was fairly constant between 40% of total occlusion and 80% of total occlusion. At 40% of total occlusion pressure, flow was about 55% of normal flow, whereas at 80% of total occlusion pressure, flow was at 49.3% of normal flow. This would indicate that there is no added advantage of increasing the restrictive pressure from 40% of total occlusion pressure any higher if blood flow remains essentially the same. This has resulted in most protocols now using 50% of total occlusion pressure as a standard BFR exercise pressure for most study designs.

The inability to set the restrictive pressures based on some standard assessment represents a significant limitation of using an elastic band or some other technique in a field setting since there is no way to reliably replicate conditions from day to day or from person to person and may result in over inflation and a possible increased risk of injury or muscle damage. One method proposed to determine the restrictive pressure that could be used during practical BFR exercise was proposed by Wilson et al. (2013) and consisted of using a perceived pain pressure scale that ranged from 0 to 10. The authors suggested that the elastic band would be tightened around the person’s limb until it resulted in a moderate pressure without causing pain. In this same study, the authors demonstrated that practical BFR exercise resulted in the elevations of muscle swelling and increased myoelectric activity, both considered indicators of muscle hypertrophy. Although Wilson’s method seemed to work as an effective approach to individualize the restrictive pressure across different individuals, it was not as precise as the method classically performed during traditional BFR exercise that utilizes Doppler ultrasound. Additionally, the reliability and reproducibility of Wilson’s scale have been recently questioned (Bell et al., 2020). Current studies are now trying to individualize the pressures to be used during practical BFR exercise based on the person’s limb circumference, the length of the elastic band, and CRTs.

For both traditional and practical BFR exercise, a typical protocol commonly consists of 4 sets of 30–15–15–15 repetitions (Wilson et al., 2013; Vechin et al., 2015). However, it should be noted that repetitions performed to failure have also been shown to be effective for inducing some neuromuscular adaptations (Sieljacks et al., 2018; Jessee et al., 2019). This approach highlights additional advantages of low-load BFR exercise over high-load resistance exercise, since there are lower mechanical stresses to the joints because of the lower loads (20% 1RM versus 70% 1RM or greater) and overall lower volume of exercise (load × total repetitions).

In terms of exercise intensity, most BFR exercise protocols include training loads between 20 and 50% 1-RM (Nakajima et al., 2010; Yasuda et al., 2010b), whereas loads higher than 50% 1-RM do not seen to provide any additional advantages (Laurentino et al., 2008). These lower loads used during BFR exercise help to lower or avoid muscle damage and soreness. Therefore, it shortens the time to recover from a single bout of exercise, potentially allowing for an increased training frequency.

The length of the rest interval between sets usually varies from 30s to 2min, with 1min and 1:30min being the most common rest periods. Two to 5min of rest have also been used between exercises for protocols that include more than 1 exercise. Traditionally, the cuffs or the elastic wraps remain inflated or tightened during the entire exercise period; in other words, the cuffs or bands are positioned immediately before the first set and removed following completion of the last set of exercise. However, due to the discomfort that BFR exercise induces, new studies have started to investigate the physiological effects of intermittent BFR exercise, in which the cuffs are deflated during the rest intervals between sets. Conflicting results have been reported so far with studies demonstrating that intermittent BFR exercise does not seem to diminish the exercise-induced physiological response (Freitas et al., 2020) while others show otherwise (Suga et al., 2010).

Regarding the muscle groups of interest, due to its the nature, BFR should be applied only to the most proximal portions of the arms and legs. For instance, even if one desires to train the calf muscles using the BFR method, the cuffs or elastic bands should still be applied to the most proximal portion of the leg and not below the knee. Finally, although the application of BFR is limited to arms and legs, previous studies have demonstrated that core muscles may also benefit indirectly from BFR exercise. For instance, Yasuda et al. (2010b) had young males complete 2weeks of twice a day bench press at 30% of 1-RM with BFR applied to the most proximal portion of both arms and observed a 6% increase bench press 1-RM strength, and 8 and 16% increases in triceps and pectoralis major muscle thicknesses, whereas no significant changes were observed for the control group following training.

## Aerobic Exercises With Blood Flow Restriction

The basic modes of exercise are either aerobic or anaerobic in nature. Aerobic exercises commonly used in combination with blood flow restriction usually include specific exercises like walking or cycling. The difficulty in setting the workload for aerobically based BFR exercises is the fact that exercise intensity is often based on some percentage of maximal heart rate (Ozaki et al., 2011a). Since blood flow restriction causes a reduction in venous return and a resultant lowering of stroke volume, the ability to maintain cardiac output during exercise is then accomplished by an increased heart rate which may not be the same cardiovascular response that would be expected based on heart rate alone if blood flow restriction was not being used (Renzi et al., 2010). Therefore, perhaps another way to set acute workloads for BFR protocols could be based on perceived exertion to the exercise intensity rather than heart rate.

Traditional aerobic exercise is well known for improving oxygen consumption and consequently endurance performance, without significantly increasing muscle hypertrophy or strength. However, studies investigating the long-term effects of aerobic exercise with BFR have demonstrated that this technique is, surprisingly, effective at enhancing neuromuscular parameters in old and young individuals.

One of the first studies investigating the long-term effects of aerobic exercise with BFR was conducted by Abe et al. (2006), who demonstrated that a walking protocol combined with BFR (160–230mmHg) twice a day over the course of 3weeks was effective at increasing muscle cross-sectional area and volume by approximately 6% each in young adults. Such increases in muscle size parameters were also accompanied by increases in maximum dynamic (i.e., 1-RM) and isometric strength. In a follow-up study from the same research group using a similar protocol, the authors were able to replicate the study results, but in this case in a cohort of older individuals aged 60 to 78years, in addition to improvements in functional parameters also being observed. In another study, Ozaki et al. (2011b) had elderly women (53–73years) complete 10weeks of walking combined with BFR, consisting of 20-min sessions performed 4 times a week, with 140mmHg to 200mmHg of BFR. After training, the authors reported significant increases in muscle cross-sectional area (~3%) and volume (2.7–3.7%) in the thigh region, as well as in isokinetic strength (~8–22%), and in functional performance measured in the timed up and go test (−10.7%), with concomitant improvements in aerobic capacity (~9%). Furthermore, these adaptations observed with aerobic exercise with BFR are not limited to walking with BFR. Abe et al. (2010a) observed significant increases in skeletal muscle size and volume in the lower body and oxygen uptake following 8weeks of cycling with BFR, although improvements in muscular strength did not reach statistical significance.

Such findings of walking in combination with BFR eliciting increases in skeletal muscle size and strength are surprising, as traditional aerobic exercise without BFR is well known for improving aerobic capacity but not skeletal muscle size or strength. Some of these increases have been reported to happen as early as after 4 consecutive training days (Abe et al., 2006). Therefore, the findings from the aforementioned studies and others have great implications for individuals unwilling or unable to perform high-intensity resistance exercise and that are seeking to improve neuromuscular parameters, as such training modality may serve as a potential training alternative to resistance training. This is particularly true for those suffering from severe sarcopenia and strength loss, such as frail older individuals and several clinical populations.

Although the findings related to aerobic exercise with BFR present significant clinical relevance, much is yet to be clarified regarding its underlying mechanisms conducing to muscle hypertrophy. The acute release of anabolic hormones has been proposed as one of the potential mechanisms, as Abe et al. (2006) detected significant increases in growth hormone immediately post- and up to 15min post-exercise, while cortisol levels remained unchanged. On the other hand, Ozaki et al. (2017) observed increases in GH in both BFR and control groups, whereas muscle hypertrophy had previously been observed only in the BFR walk group (Ozaki et al., 2011b). Additionally, there is an intense debate on the contributions of anabolic hormones to the exercise-induced hypertrophic response (Schroeder et al., 2013). It has also been speculated that a potential exercise-induced metabolic response during aerobic exercise with BFR could facilitate the recruitment of the more prone to hypertrophy type II muscle fibers. However, the metabolic response to walking with BFR is either minimal or non-existent according to Loenneke et al. (2012d), although a more pronounced increase was observed by Ozaki et al. (2014). Another suggested mechanism through each aerobic exercise with BFR elicits its positive neuromuscular adaptations is the exercise-induced muscle swelling. The muscle swelling response has long been thought to be one of the mechanisms leading to muscle hypertrophy in the context of resistance exercise with BFR (Loenneke et al., 2012c). Although little is known regarding the effects of aerobic exercise with BFR on muscle swelling, specially concerning walking, Ogawa et al. (2012) reported significant percent change increases in muscle thickness immediately post a single bout of walking with BFR. Lastly, the modulation of biomolecular pathways governing muscle protein turnover, such as Akt/mTOR and myostatin, has also been speculated to contribute to the reported responses to aerobic exercise with BFR. Ozaki et al. (2014) reported significant increases in phosphorylation of Erk1/2 and p38 following a walking protocol with BFR; however, it should be highlighted that increases in p38 were also observed in the non-BFR walking condition; additionally, eEF2 phosphorylation was lower for BFR walking compared to non-BFR walking, no significant changes were observed in Akt and mTOR phosphorylation levels, as well as no differences between conditions for changes in SK1 phosphorylation, and eEF2 phosphorylation was lower for the BFR condition. Nonetheless, these results should be interpreted with caution due to the limited samples size (i.e., 6 participants) and limitations of the study design. Therefore, additional studies are critically needed to further elucidate the mechanism through which aerobic exercise combined with BFR induces muscle hypertrophy.

## Anaerobic Exercises With Blood Flow Restriction

For anaerobic-based modalities, that is, resistance exercises, intensities most often will be based on relative loads (percent of maximal strength, % 1RM) rather than absolute loads (Giles et al., 2017). It should be noted that several modes of contraction exist to assess performances to resistance exercise which include isometric contractions (no visible movement of the limb during muscular contraction), isotonic contractions (constant force being generated throughout the entire range of motion as controlled devices like Cybex or KinCom), isokinetic contractions (constant velocity of movement throughout the entire range of motion as controlled by devices like Cybex or KinCom), or dynamic contractions with the use of free weights. In many studies, relative loads of 20 to 30% 1RM with blood flow restriction have been compared to traditional high-intensity (80% 1RM; Laurentino et al., 2008, 2012; Karabulut et al., 2010) resistance training without blood flow restriction but blood flow restriction exercise loads have ranged from 20 to 50% 1RM.

As mentioned above, the original protocols designed for the KAATSU-Master and BFR resistance exercise used loads of 20 to 30% 1RM with 4 sets of exercises composed of 1 set of 30 repetitions followed by 3 sets of 15 repetitions with 1-min rest periods between sets and at a cadence of 1.5s in both the eccentric and concentric portions of the movement (Freitas et al., 2017; Miller et al., 2018). When multiple exercises are used (usually 2 or 3 total exercises for a given session, like 2 leg press, followed knee extension, and knee flexion), then rest periods between exercises are usually between 3 and 5min. Normally, the restrictive pressures would be maintained throughout the entire session but recent studies indicate that releasing the pressure in the cuffs between different muscle groups does not seem to diminish the effects of the exercise protocol and are as effective as the continuous restriction protocols (Beaven et al., 2012; Yasuda et al., 2013). Many of the early studies used a single, acute bout of the exercise to explore various physiological responses and then imply that the lower intensity protocols used with blood flow restriction (20–30% 1RM) were as effective as high-intensity (80% 1RM) resistance exercise for gaining strength and promoting muscle hypertrophy. Training protocols using blood flow restriction are fairly limited and usually short in duration, normally lasting between 4 and 8weeks (Clark et al., 2011; Cook et al., 2014; Conceição et al., 2018; Held et al., 2020; Karabulut et al., 2020; Zhao et al., 2020). Most often, training protocols use 3 training sessions per week with each session separated by 48h (Clark et al., 2011; Held et al., 2020) but some designs use daily bouts of exercise (5 times per week) and some even use 2 bouts per day for 8 to 10 consecutive days (Iida et al., 2006).

## Limitations From Previous Studies and Current Gaps in the Research Literature

The ability to accurately interpret results from a published paper is often dependent on the clarity of the research design, participant and protocol descriptions, and the clear description of the statistics used to analyze the data. As with most research designs, there are an infinite number of combinations of potentially confounding issues related to participant selection for the protocol, like age, sex, training status, health status, nutritional status, hormonal status, etc., but with blood flow restriction protocols, many other factors must also be considered. These additional factors could include the type of cuff used to induce the restriction pressure (width of the cuff, type of material, pneumatically controlled cuffs or tension wraps or bandages applied based on perceived discomfort; Loenneke et al., 2014c; Buckner et al., 2017; Stray-Gundersen et al., 2020), initial pressure/tightness of the cuffs before inflation (Karabulut et al., 2011a, 2014), the restrictive pressure used (absolute, relative, percent of total restrictive pressure, intermittent pressure, continuous pressure; Murray et al., 2020), the composition (fat and muscle mass; Karabulut et al., 2014) and size (circumference or girth) of the limb being restricted (Loenneke et al., 2014a), the mode of exercise (walking, cycling, resistance training, absolute loads, relative loads, contraction types – isometric, isotonic, isokinetic), the protocols used if exercise is required (number of repetitions, number of sets, muscle groups, cadence of the concentric and eccentric portions of the movement, time under tension), and if the responses are acute or due to prolonged training (how many sessions, days, weeks, etc.).

Over the past 20years, most blood flow restriction studies have used male subjects, often college-aged males (18–25years of age) because of convenience; however, a few studies have also included college-aged females but then failed to separate the sexes when analyzing the data. Only recently, a few studies have focused solely on female participants (Loenneke et al., 2014a). The importance of separating the responses based on sex is grounded in the differences in hormonal status between men and women (testosterone levels, estrogen levels, phase of the menstrual cycle, etc.). As mentioned earlier, many studies have used a college-aged population (Abe et al., 2010a), but some studies have also investigated middle-aged (35–55years of age) and older subjects (over age 65years; Abe et al., 2010b; Ozaki et al., 2011a,b), but these studies are much fewer in number.

The training status of participants has not always been reported in many BFR exercise studies, making the interpretation of the results difficult since the more sedentary or deconditioned the subject, the greater the magnitude of change that might be expected. Obviously, health status will also affect the outcome measures of any study, with normal healthy individuals responding very differently than those that have a compromised health status or those having a clinically diagnosed condition like hypertension, diabetes, multiple sclerosis, osteoarthritis, etc. It is also important to examine the nutritional status of participants in research studies since certain supplements (creatine, protein, etc.) or diets deficient in some nutrients (protein, vitamins, etc.) could also affect outcome variables that are being assessed following BFR protocols, like muscle growth or hypertrophy and improvements in muscle strength.

When examining cuff type, factors like cuff width, the material of the cuff, and whether the restriction pressure is carefully set and controlled with a pneumatic device in a laboratory setting or if the pressure is not known because a practical elastic wrap has been used in a community-based setting, also needs to be considered and reported in the literature. The importance of reporting cuff width is critical since the amount of occlusion increases as the cuff width increases at similar restrictive pressures (Loenneke et al., 2012b) resulting in increased brachial and central blood pressures, heart rates, perceived efforts, and perceived pain. The original KAATSU-Master uses an elastic 3cm wide cuff for the arms and a 5cm cuff for the legs, while the Hokanson pneumatic device uses a nylon 3.5cm cuff for the arms and a 13.5cm nylon cuff for the legs. In general, cuff widths have ranged from 2cm for the arms to over 20cm for the legs.

The material of the cuff is also important to report. In general, the differences between elastic and nylon cuffs are minimal with both types resulting in similar numbers of repetitions to fatigue and similar ratings of perceived exertion and discomfort; however, some reports indicate that the arterial occlusion pressures were significantly greater when using the elastic cuff when compared to the nylon cuff. The major issue with elastic wraps (i.e., tensor bandages) used in a community setting is the issue that there is no way to monitor or assess the exact level of occlusion that is occurring since the tightness of the wraps will determine the amount of restriction, so unless a person is very experienced with the technique of blood flow restriction exercise and is very aware of the sensation that should be felt with appropriate restriction, it is generally recommended that the use of elastic wraps should not be used in gym settings because of the tendency to over restrict blood flow or to inadvertently totally occlude blood flow altogether.

The original research that used the KAATSU-Master device used a restrictive pressure of 120% of upper body SBP (Arm SBP×1.2) for the arms (normally around 140–160mmHg) and then added an additional 20% to the 120% of upper body SBP cuff pressure (Arm SBP×1.44) to account for differences in lower body SBP for the legs (usually around 180–240mmHg). This original research also maintained the constant restrictive pressure for the entire exercise session across all repetitions, sets, and muscle groups, and the pressures were the same for all participants regardless of the size of the limbs that were being exercised. More recent studies have explored the ability to release the restrictive pressure in the cuffs between different muscle groups (after 2 leg press and before knee extensions for example) and have reported similar results to protocols that use pressures that are continuously applied throughout the session (Burgomaster et al., 2003). Also, based on the concept of individualization, researchers have now been determining the pressures needed to totally occlude the legs individually or the arms individually, and then using some relative percentage of the total occlusion pressure for the exercise session (Laurentino et al., 2008). These relative pressures have been around 50% of the pressure needed for total occlusion with the idea that this would result in 50% of the blood flow to the exercising limb would also be restricted; however, studies have indicated that blood flow to the restricted limb (about 50% of normal flow without restriction) is similar for restrictive pressures between 40 and 90% of total occlusion pressure for a given limb when utilizing techniques that can actually measure blood flow, like Doppler ultrasound (Counts et al., 2016).

The concept that limb composition might have an effect of the amount of blood flow restriction that a limb might experience depending on the pressure being used is based on the ability to compress the different tissues of the limb (fat versus muscle). It is logical to think that limbs containing more fat might need more pressure to achieve the desired restriction to blood flow since it was thought that fat would compress and absorb most the pressure without compressing the vasculature that would be closer to the muscle. One study (Karabulut et al., 2011a) reported that thigh composition and size had a significant impact on the effects of initial restrictive pressure. Other studies have investigated the amounts of fat and muscle mass in the limb being restricted and have reported that the circumference of the limb is more predictive of restrictive pressure as compared to the composition of the limb with larger limbs needing greater pressures to achieve a given level of blood flow restriction (Loenneke et al., 2014a).

## Conclusion

Blood flow restriction exercise remains a relatively safe training strategy for those unable or unwilling to perform high-intensity resistance exercise or endurance exercise, yet wanting to improve neuromuscular parameters, such as muscular strength, power, and function, and improve aerobic endurance. However, considering the complexity of the technique, questions still need to be answered regarding the precise underlying mechanisms responsible for the adaptations to BFR exercise, as well as practical concerns, such as determining the most appropriate restrictive pressures to be applied, the type of restrictive devices to be used, potential risks for clinical populations – especially concerning the cardiovascular system and adequate lengths of time for training and the volumes of low-intensity exercise that need to be performed.

## Author Contributions

All authors contributed equally to writing and proofreading the manuscript, and also approved the content of the manuscript’s final version.

## Conflict of Interest

The authors declare that the research was conducted in the absence of any commercial or financial relationships that could be construed as a potential conflict of interest.

## Publisher’s Note

All claims expressed in this article are solely those of the authors and do not necessarily represent those of their affiliated organizations, or those of the publisher, the editors and the reviewers. Any product that may be evaluated in this article, or claim that may be made by its manufacturer, is not guaranteed or endorsed by the publisher.

## References

[ref1] Abe T. Fujita S. Nakajima T. Sakamaki M. Ozaki H. Oga-R.. (2010a). Effects of low-intensity cycle training with restricted leg blood flow on thigh muscle volume and VO 2max in young men. J. Sports Sci. Med. 9, 452–458. PMID: 24149640 PMC3761718

[ref2] Abe T. Kearns C. F. Sato Y. (2006). Muscle size and strength are increased following walk training with restricted venous blood flow from the leg muscle, Kaatsu-walk training. J. Appl. Physiol. 100, 1460–1466. doi: 10.1152/japplphysiol.01267.2005, PMID: 16339340

[ref3] Abe T. Sakamaki M. Fujita S. Ozaki H. Sugaya M. Sato Y.. (2010b). Effects of low-intensity walk training with restricted leg blood flow on muscle strength and aerobic capacity in older adults. J. Geriatr. Phys. Ther. 33, 34–40. doi: 10.1152/japplphysiol.01267.2005, PMID: 20503732

[ref4] Alvarez I. F. Damas F. de Biazon T. M. P. Miquelini M. Doma K. Libardi C. A. (2019). Muscle damage responses to resistance exercise performed with high-load versus low-load associated with partial blood flow restriction in young women. Eur. J. Sport Sci. 20, 125–134. doi: 10.1080/17461391.2019.1614680, PMID: 31043129

[ref5] Amani-Shalamzari S. Rajabi S. Rajabi H. Gahreman D. E. Paton C. Bayati M.. (2019). Effects of blood flow restriction and exercise intensity on aerobic, anaerobic, and muscle strength adaptations in physically active collegiate women. Front. Physiol. 10:810. doi: 10.3389/fphys.2019.00810, PMID: 31297065 PMC6607282

[ref6] Amano S. Ludin A. F. M. Clift R. Nakazawa M. Law T. D. Rush L. J.. (2016). Effectiveness of blood flow restricted exercise compared with standard exercise in patients with recurrent low back pain: study protocol for a randomized controlled trial. Trials 17:81. doi: 10.1186/s13063-016-1214-7, PMID: 26867541 PMC4751635

[ref7] Anderson B. Kelly A.-M. Kerr D. Clooney M. Jolley D. (2008). Impact of patient and environmental factors on capillary refill time in adults. Am. J. Emerg. Med. 26, 62–65. doi: 10.1016/j.ajem.2007.06.026, PMID: 18082783

[ref8] Beaven C. M. Cook C. J. Kilduff L. Drawer S. Gill N. (2012). Intermittent lower-limb occlusion enhances recovery after strenuous exercise. Appl. Physiol. Nutr. Metab. 37, 1132–1139. doi: 10.1139/h2012-101, PMID: 22970789

[ref9] Bell Z. W. Dankel S. J. Spitz R. W. Chatakondi R. N. Abe T. Loenneke J. P. (2020). The perceived tightness scale does not provide reliable estimates of blood flow restriction pressure. J. Sport Rehabil. 29, 516–518. doi: 10.1123/jsr.2018-0439, PMID: 31553951

[ref10] Bjørnsen T. Wernbom M. Løvstad A. Paulsen G. D’Souza R. F. Cameron-Smith D.. (2019). Delayed myonuclear addition, myofiber hypertrophy, and increases in strength with high-frequency low-load blood flow restricted training to volitional failure. J. Appl. Physiol. 126, 578–592. doi: 10.1152/japplphysiol.00397.2018, PMID: 30543499

[ref11] Brandner C. R. Kidgell D. J. Warmington S. A. (2014). Unilateral bicep curl hemodynamics: low-pressure continuous vs high-pressure intermittent blood flow restriction. Scand. J. Med. Sci. Sports 25, 770–777. doi: 10.1111/sms.12297, PMID: 25055880

[ref12] Buckner S. L. Dankel S. J. Counts B. R. Jessee M. B. Mouser J. G. Mattocks K. T.. (2017). Influence of cuff material on blood flow restriction stimulus in the upper body. J. Physiol. Sci. 67, 207–215. doi: 10.1007/s12576-016-0457-0, PMID: 27194224 PMC10717541

[ref13] Burgomaster K. A. Moore D. R. Schofield L. M. Phillips S. M. Sale D. G. Gibala M. J. (2003). Resistance training with vascular occlusion: metabolic adaptations in human muscle. Med. Sci. Sports Exerc. 35, 1203–1208. doi: 10.1249/01.MSS.0000074458.71025.71, PMID: 12840643

[ref14] Burr J. F. Hughes L. Warmington S. Scott B. R. Owens J. Abe T.. (2020). Response: commentary: can blood flow restricted exercise cause muscle damage? Commentary on blood flow restriction exercise: considerations of methodology, application, and safety. Front. Physiol. 11:574633. doi: 10.3389/fphys.2020.574633, PMID: 33192577 PMC7662128

[ref15] Clark B. C. Manini T. M. Hoffman R. L. Williams P. S. Guiler M. K. Knutson M. J.. (2011). Relative safety of 4 weeks of blood flow-restricted resistance exercise in young, healthy adults. Scand. J. Med. Sci. Sports 21, 653–662. doi: 10.1111/j.1600-0838.2010.01100.x, PMID: 21917016 PMC6152804

[ref160] Conceição M. S. Junior E. M. Telles G. D. Libardi C. A. Castro A. Andrade A. L. L.. (2019). Augmented anabolic responses after 8-wk cycling with blood flow restriction. Med. Sci. Sports Exerc. 51, 84–93. doi: 10.1249/MSS.0000000000001755, PMID: 30113523

[ref17] Cook C. J. Kilduff L. P. Beaven C. M. (2014). Improving strength and power in trained athletes with 3 weeks of occlusion training. Int. J. Sports Physiol. Perform. 9, 166–172. doi: 10.1123/ijspp.2013-0018, PMID: 23628627

[ref18] Counts B. R. Dankel S. J. Barnett B. E. Kim D. Mouser J. G. Allen K. M.. (2016). Influence of relative blood flow restriction pressure on muscle activation and muscle adaptation. Muscle Nerve 53, 438–445. doi: 10.1002/mus.24756, PMID: 26137897

[ref19] Counts B. R. Rossow L. M. Mattocks K. T. Mouser J. G. Jessee M. B. Buckner S. L.. (2018). Let’s talk about sex: where are the young females in blood flow restriction research? Clin. Physiol. Funct. Imaging 38, 1–3. doi: 10.1111/cpf.12394, PMID: 27730736

[ref20] Fahs C. A. Rossow L. M. Loenneke J. P. Thiebaud R. S. Kim D. Bemben D. A.. (2012). Effect of different types of lower body resistance training on arterial compliance and calf blood flow. Clin. Physiol. Funct. Imaging 32, 45–51. doi: 10.1111/j.1475-097X.2011.01053.x, PMID: 22152078

[ref21] Farell P. A. Joyner M. J. Caiozzo V. J. (2011). ACSM’s advanced exercise physiology. Wolters Kluwer Health Adis (ESP).

[ref22] Fleming S. Gill P. Jones C. Taylor J. A. Van den Bruel A. Heneghan C.. (2015). The diagnostic value of capillary refill time for detecting serious illness in children: a systematic review and meta-analysis. PLoS One 10:e0138155. doi: 10.1371/journal.pone.0138155, PMID: 26375953 PMC4573516

[ref23] Fleming S. Gill P. J. Van den Bruel A. Thompson M. (2016). Capillary refill time in sick children: a clinical guide for general practice. Br. J. Gen. Pract. 66:587. doi: 10.3399/bjgp16X687925, PMID: 27789509 PMC5072914

[ref24] Freitas E. D. S. Bemben M. G. Silva A. S. Aniceto R. R. Ferreira-Junior J. B. Cirilo-Sousa M. S. (2017). Resistance exercise performed at different degrees of arterial occlusion pressure does not induce prolonged oxidative stress or muscle damage. Int. J. Sports Exerc. Med. 3, 1–9. doi: 10.23937/2469-5718/1510075

[ref25] Freitas E. D. S. Miller R. M. Heishman A. D. Ferreira-Júnior J. B. Araújo J. P. Bemben M. G. (2020). Acute physiological responses to resistance exercise with continuous versus intermittent blood flow restriction: a randomized controlled trial. Front. Physiol. 11:132. doi: 10.3389/fphys.2020.00132, PMID: 32256374 PMC7090220

[ref26] Fry C. S. Glynn E. L. Drummond M. J. Timmerman K. L. Fujita S. Abe T.. (2010). Blood flow restriction exercise stimulates mTORC1 signaling and muscle protein synthesis in older men. J. Appl. Physiol. 108, 1199–1209. doi: 10.1152/japplphysiol.01266.2009, PMID: 20150565 PMC2867530

[ref27] Fullana J. M. Cros F. Becker F. Ouchene A. Partsch H. (2005). The venous return simulator: an effective tool for investigating the effects of external compression on the venous hemodynamics–first results after thigh compression. Vasa 34, 19–23. doi: 10.1024/0301-1526.34.1.19, PMID: 15786933

[ref28] Gallier J. (2020). How to measure capillary refill time in patient’s who are acutely ill. Nurs. Times 116, 29–30.

[ref29] Giles L. Webster K. E. McClelland J. Cook J. L. Giles I. L. (2017). Quadriceps strengthening with and without blood flow restriction in the treatment of patellofemoral pain: a double-blind randomised trial. Br. J. Sports Med. 51, 1688–1694. doi: 10.1136/bjsports-2016-096329, PMID: 28500081

[ref30] Goldfarb A. H. Garten R. S. Chee P. D. M. Cho C. Reeves G. V. Hollander D. B.. (2008). Resistance exercise effects on blood glutathione status and plasma protein carbonyls: influence of partial vascular occlusion. Eur. J. Appl. Physiol. 104, 813–819. doi: 10.1007/s00421-008-0836-1, PMID: 18661144

[ref31] Goto K. Ishii N. Kizuka T. Takamatsu K. (2005). The impact of metabolic stress on hormonal responses and muscular adaptations. Med. Sci. Sports Exerc. 37, 955–963. doi: 10.1249/01.mss.0000170470.98084.39, PMID: 15947720

[ref32] Held S. Behringer M. Donath L. (2020). Low intensity rowing with blood flow restriction over 5 weeks increases V̇O2max in elite rowers: a randomized controlled trial. J. Sci. Med. Sport 23, 304–308. doi: 10.1016/j.jsams.2019.10.002, PMID: 31672481

[ref33] Iida H. Kurano M. Takano H. Oonuma H. Imuta H. Kubota N.. (2006). Can KAATSU be used for an orthostatic stress in astronauts?: a case study. Int. J. KAATSU Train. Res. 2, 45–52. doi: 10.3806/ijktr.2.45

[ref34] Jessee M. B. Buckner S. L. Mattocks K. T. Dankel S. J. Mouser J. G. Bell Z. W.. (2019). Blood flow restriction augments the skeletal muscle response during very low-load resistance exercise to volitional failure. Physiol. Int. 106, 180–193. doi: 10.1556/2060.106.2019.15, PMID: 31262205

[ref35] Jørgensen S. L. Bohn M. B. Aagaard P. Mechlenburg I. (2020). Efficacy of low-load blood flow restricted resistance EXercise in patients with knee osteoarthritis scheduled for total knee replacement (EXKnee): protocol for a multicentre randomised controlled trial. BMJ Open 10:e034376. doi: 10.1136/bmjopen-2019-034376, PMID: 33004382 PMC7534706

[ref36] Kambič T. Novaković M. Tomažin K. Strojnik V. Božič-Mijovski M. Jug B. (2021). Hemodynamic and hemostatic response to blood flow restriction resistance exercise in coronary artery disease. J. Cardiovasc. Nurs. 36, 507–516. doi: 10.1097/JCN.0000000000000699, PMID: 32496365

[ref37] Kambič T. Novaković M. Tomažin K. Strojnik V. Jug B. (2019). Blood flow restriction resistance exercise improves muscle strength and hemodynamics, but not vascular function in coronary artery disease patients: a pilot randomized controlled trial. Front. Physiol. 10:656. doi: 10.3389/fphys.2019.00656, PMID: 31244668 PMC6581774

[ref38] Karabulut M. Abe T. Sato Y. Bemben M. G. (2010). The effects of low-intensity resistance training with vascular restriction on leg muscle strength in older men. Eur. J. Appl. Physiol. 108, 147–155. doi: 10.1007/s00421-009-1204-5, PMID: 19760431

[ref39] Karabulut M. Bemben D. A. Sherk V. D. Anderson M. A. Abe T. Bemben M. G. (2011a). Effects of high-intensity resistance training and low-intensity resistance training with vascular restriction on bone markers in older men. Eur. J. Appl. Physiol. 111, 1659–1667. doi: 10.1007/s00421-010-1796-9, PMID: 21207053

[ref40] Karabulut U. Karabulut M. James E. G. (2018). Small arteries stay stiff for a longer period following vibration exercises in combination with blood flow restriction. Clin. Physiol. Funct. Imaging 38, 1000–1007. doi: 10.1111/cpf.12516, PMID: 29618165

[ref41] Karabulut M. Leal J. A. Garcia S. D. Cavazos C. Bemben M. (2014). Tissue oxygenation, strength and lactate response to different blood flow restrictive pressures. Clin. Physiol. Funct. Imaging 34, 263–269. doi: 10.1111/cpf.12090, PMID: 24119192

[ref42] Karabulut M. Lopez J. A. Karabulut U. (2020). Aerobic training session length affects arterial elasticity. Clin. Physiol. Funct. Imaging 40, 14–20. doi: 10.1111/cpf.12596, PMID: 31552704

[ref43] Karabulut M. McCarron J. Abe T. Sato Y. Bemben M. (2011b). The effects of different initial restrictive pressures used to reduce blood flow and thigh composition on tissue oxygenation of the quadriceps. J. Sports Sci. 29, 951–958. doi: 10.1080/02640414.2011.572992, PMID: 21547832

[ref44] Karabulut M. Sherk V. D. Bemben D. A. Bemben M. G. (2013). Inflammation marker, damage marker and anabolic hormone responses to resistance training with vascular restriction in older males. Clin. Physiol. Funct. Imaging 33, 393–399. doi: 10.1111/cpf.12044, PMID: 23701309

[ref45] Kilgas M. A. Lytle L. L. M. Drum S. N. Elmer S. J. (2019). Exercise with blood flow restriction to improve quadriceps function long after ACL reconstruction. Int. J. Sports Med. 40, 650–656. doi: 10.1055/a-0961-1434, PMID: 31342480

[ref46] Korakakis V. Whiteley R. Giakas G. (2018). Low load resistance training with blood flow restriction decreases anterior knee pain more than resistance training alone. A pilot randomised controlled trial. Phys. Ther. Sport 34, 121–128. doi: 10.1016/j.ptsp.2018.09.007, PMID: 30268966

[ref47] Laurentino G. C. Loenneke J. P. Mouser J. G. Buckner S. L. Counts B. R. Dankel S. J.. (2018). Validity of the handheld Doppler to determine lower-limb blood flow restriction pressure for exercise protocols. J. Strength Cond. Res. 34, 2693–2696. doi: 10.1519/JSC.0000000000002665, PMID: 29912080

[ref48] Laurentino G. Ugrinowitsch C. Aihara A. Y. Fernandes A. R. Parcell A. C. Ricard M.. (2008). Effects of strength training and vascular occlusion. Int. J. Sports Med. 29, 664–667. doi: 10.1055/s-2007-989405, PMID: 18213536

[ref49] Laurentino G. C. Ugrinowitsch C. Roschel H. Aoki M. S. Soares A. G. Neves M.. (2012). Strength training with blood flow restriction diminishes myostatin gene expression. Med. Sci. Sports Exerc. 44, 406–412. doi: 10.1249/MSS.0b013e318233b4bc, PMID: 21900845

[ref50] Loenneke J. P. Abe T. (2012). Does blood flow restricted exercise result in prolonged torque decrements and muscle damage? Eur. J. Appl. Physiol. 112, 3445–3449. doi: 10.1007/s00421-012-2312-1, PMID: 22246024

[ref51] Loenneke J. P. Allen K. M. Mouser J. G. Thiebaud R. S. Kim D. Abe T.. (2014a). Blood flow restriction in the upper and lower limbs is predicted by limb circumference and systolic blood pressure. Eur. J. Appl. Physiol. 115, 397–405. doi: 10.1007/s00421-014-3030-7, PMID: 25338316

[ref52] Loenneke J. P. Fahs C. A. A. Rossow L. M. M. Abe T. Bemben M. G. G. (2012a). The anabolic benefits of venous blood flow restriction training may be induced by muscle cell swelling. Med. Hypotheses 78, 151–154. doi: 10.1016/j.mehy.2011.10.014, PMID: 22051111

[ref53] Loenneke J. P. Fahs C. A. Rossow L. M. Sherk V. D. Thiebaud R. S. Abe T.. (2012b). Effects of cuff width on arterial occlusion: implications for blood flow restricted exercise. Eur. J. Appl. Physiol. 112, 2903–2912. doi: 10.1007/s00421-011-2266-8, PMID: 22143843 PMC4133131

[ref54] Loenneke J. Fahs C. Thiebaud R. Rossow L. Abe T. Ye X.. (2012c). The acute muscle swelling effects of blood flow restriction. Acta Physiol. Hung. 99, 400–410. doi: 10.1556/APhysiol.99.2012.4.4, PMID: 23238542

[ref55] Loenneke J. P. Kearney M. L. Thrower A. D. Collins S. Pujol T. J. (2010). The acute response of practical occlusion in the knee extensors. J. Strength Cond. Res. 24, 2831–2834. doi: 10.1519/JSC.0b013e3181f0ac3a, PMID: 20885201

[ref56] Loenneke J. P. Thiebaud R. S. Abe T. (2014b). Does blood flow restriction result in skeletal muscle damage? A critical review of available evidence. Scand. J. Med. Sci. Sports 24, e415–e422. doi: 10.1111/sms.12210, PMID: 24650102

[ref57] Loenneke J. P. Thiebaud R. S. Fahs C. A. Rossow L. M. Abe T. Bemben M. G. (2014c). Blood flow restriction: effects of cuff type on fatigue and perceptual responses to resistance exercise. Acta Physiol. Hung. 101, 158–166. doi: 10.1556/APhysiol.101.2014.2.4, PMID: 24901077

[ref58] Loenneke J. P. Thrower A. D. Balapur A. Barnes J. T. Pujol T. J. (2012d). Blood flow-restricted walking does not result in an accumulation of metabolites. Clin. Physiol. Funct. Imaging 32, 80–82. doi: 10.1111/j.1475-097X.2011.01059.x, PMID: 22152083

[ref59] McCully K. K. Smith S. Rajaei S. Leigh J. S. Natelson B. H. (2004). Muscle metabolism with blood flow restriction in chronic fatigue syndrome. J. Appl. Physiol. 96, 871–878. doi: 10.1152/japplphysiol.00141.2003, PMID: 14578362 PMC2680353

[ref60] McEwen J. A. Owens J. G. Jeyasurya J. (2019). Why is it crucial to use personalized occlusion pressures in blood flow restriction (BFR) rehabilitation? J. Med. Biol. Eng. 39, 173–177. doi: 10.1007/s40846-018-0397-7

[ref61] Miller R. M. Keeter V. M. Freitas E. D. S. Heishman A. D. Knehans A. W. Bemben D. A.. (2018). Effects of blood-flow restriction combined with postactivation potentiation stimuli on jump performance in recreationally active men. J. Strength Cond. Res. 32, 1869–1874. doi: 10.1519/JSC.0000000000002110, PMID: 28682937

[ref62] Mouser J. G. Ade C. J. Black C. D. Bemben D. A. Bemben M. G. (2017). Brachial blood flow under relative levels of blood flow restriction is decreased in a nonlinear fashion. Clin. Physiol. Funct. Imaging 38, 425–430. doi: 10.1111/cpf.12432, PMID: 28402045

[ref63] Mouser G. J. Mattocks K. T. Dankel S. J. Buckner S. L. Jessee M. B. Bell Z. W.. (2018). Very low load resistance exercise in the upper body with and without blood flow restriction: cardiovascular outcomes. Appl. Physiol. Nutr. Metab. 44, 288–292. doi: 10.1139/apnm-2018-0325, PMID: 30148969

[ref64] Murray J. Bennett H. Boyle T. Williams M. Davison K. (2020). Approaches to determining occlusion pressure for blood flow restricted exercise training: systematic review. J. Sports Sci. 39, 663–672. doi: 10.1080/02640414.2020.1840734, PMID: 33135570

[ref65] Nakajima T. Kurano M. Iida H. Takano H. Oonuma H. Morita T.. (2006). Use and safety of KAATSU training: results of a national survey. Int. J. KAATSU Train. Res. 2, 5–13. doi: 10.3806/ijktr.2.5

[ref66] Nakajima T. Kurano M. Sakagami F. Iida H. Fukumura K. Fukuda T.. (2010). Effects of low-intensity KAATSU resistance training on skeletal muscle size/strength and endurance capacity in patients with ischemic heart disease. Int. J. KAATSU Train. Res. 6, 1–7. doi: 10.3806/ijktr.6.1

[ref67] Nyakayiru J. Fuchs C. J. Trommelen J. Smeets J. S. J. Senden J. M. Gijsen A. P.. (2019). Blood flow restriction only increases myofibrillar protein synthesis with exercise. Med. Sci. Sports Exerc. 51, 1137–1145. doi: 10.1249/MSS.0000000000001899, PMID: 30694972 PMC6553970

[ref68] Ogawa M. Loenneke J. P. Yasuda T. Fahs C. A. Lindy M. Thiebaud R. S.. (2012). Time course changes in muscle size and fatigue during walking with restricted leg blood flow in young men. J. Phys. Educ. Sport Manag. 3, 14–19. doi: 10.5897/JPESM12.002

[ref69] Ozaki H. Kakigi R. Kobayashi H. Loenneke J. P. Abe T. Naito H. (2014). Effects of walking combined with restricted leg blood flow on mTOR and MAPK signalling in young men. Acta Physiol. 211, 97–106. doi: 10.1111/apha.12243, PMID: 24479982

[ref70] Ozaki H. Loenneke J. P. Abe T. (2017). Blood flow-restricted walking in older women: does the acute hormonal response associate with muscle hypertrophy? Clin. Physiol. Funct. Imaging 37, 379–383. doi: 10.1111/cpf.12312, PMID: 26517965

[ref71] Ozaki H. Miyachi M. Nakajima T. Abe T. (2011a). Effects of 10 weeks walk training with leg blood flow reduction on carotid arterial compliance and muscle size in the elderly adults. Angiology 62, 81–86. doi: 10.1177/0003319710375942, PMID: 20682613

[ref72] Ozaki H. Sakamaki M. Yasuda T. Fujita S. Ogasawara R. Sugaya M.. (2011b). Increases in thigh muscle volume and strength by walk training with leg blood flow reduction in older participants. J. Gerontol. A Biol. Sci. Med. Sci. 66, 257–263. doi: 10.1093/gerona/glq182, PMID: 20974731

[ref73] Petrick H. L. Pignanelli C. Barbeau P. A. Churchward-Venne T. A. Dennis K. M. J. H. van Loon L. J. C.. (2019). Blood flow restricted resistance exercise and reductions in oxygen tension attenuate mitochondrial H2O2 emission rates in human skeletal muscle. J. Physiol. 15, 3985–3997. doi: 10.1113/JP277765, PMID: 31194254

[ref74] Pinto R. R. Karabulut M. Poton R. Polito M. D. (2018). Acute resistance exercise with blood flow restriction in elderly hypertensive women: haemodynamic, rating of perceived exertion and blood lactate. Clin. Physiol. Funct. Imaging 38, 17–24. doi: 10.1111/cpf.12376, PMID: 27283375

[ref75] Renzi C. P. Tanaka H. Sugawara J. (2010). Effects of leg blood flow restriction during walking on cardiovascular function. Med. Sci. Sports Exerc. 42, 726–732. doi: 10.1249/MSS.0b013e3181bdb454, PMID: 19952840 PMC2888901

[ref76] Sato Y. (2005). The history and future of KAATSU training. Int. J. KAATSU Train. Res. 1, 1–5. doi: 10.3806/ijktr.1.1

[ref77] Sato Y. Yoshitomi A. Abe T. (2005). Acute growth hormone response to low-intensity KAATSU resistance exercise: comparison between arm and leg. Int. J. KAATSU Train. Res. 1, 45–50. doi: 10.3806/ijktr.1.45

[ref78] Schriger D. L. Baraff L. (1988). Defining normal capillary refill: variation with age, sex, and temperature. Ann. Emerg. Med. 17, 932–935. doi: 10.1016/S0196-0644(88)80675-9, PMID: 3415066

[ref79] Schroeder E. T. Villanueva M. West D. D. W. Phillips S. M. (2013). Are acute post-resistance exercise increases in testosterone, growth hormone, and IGF-1 necessary to stimulate skeletal muscle anabolism and hypertrophy? Med. Sci. Sports Exerc. 45, 2044–2051. doi: 10.1249/MSS.0000000000000147, PMID: 24136137

[ref80] Sieljacks P. Degn R. Hollaender K. Wernbom M. Vissing K. (2018). Non-failure blood flow restricted exercise induces similar muscle adaptations and less discomfort than failure protocols. Scand. J. Med. Sci. Sports 29, 336–347. doi: 10.1111/sms.13346, PMID: 30475424

[ref81] Silverstein D. Hopper K. (2014). Small animal critical care medicine-E-Book. Elsevier Health Sciences.

[ref82] Spranger M. D. Krishnan A. C. Levy P. D. O’Leary D. S. Smith S. A. (2015). Blood flow restriction training and the exercise pressor reflex: a call for concern. Am. J. Physiol. Heart Circ. Physiol. 309, H1440–H1452. doi: 10.1152/ajpheart.00208.2015, PMID: 26342064 PMC7002872

[ref83] Stray-Gundersen S. Wooten S. Tanaka H. (2020). Walking with leg blood flow restriction: wide-rigid cuffs vs. narrow-elastic bands. Front. Physiol. 11:568. doi: 10.3389/fphys.2020.00568, PMID: 32547424 PMC7273976

[ref84] Sudo M. Ando S. Kano Y. (2017). Repeated blood flow restriction induces muscle fiber hypertrophy. Muscle Nerve 55, 274–276. doi: 10.1002/mus.25415, PMID: 27668404

[ref85] Suga T. Okita K. Morita N. Yokota T. Hirabayashi K. Horiuchi M.. (2009). Intramuscular metabolism during low-intensity resistance exercise with blood flow restriction. J. Appl. Physiol. 106, 1119–1124. doi: 10.1152/japplphysiol.90368.2008, PMID: 19213931

[ref86] Suga T. Okita K. Morita N. Yokota T. Hirabayashi K. Horiuchi M.. (2010). Dose effect on intramuscular metabolic stress during low-intensity resistance exercise with blood flow restriction. J. Appl. Physiol. 108, 1563–1567. doi: 10.1152/japplphysiol.00504.2009, PMID: 20360434

[ref87] Suga T. Okita K. Takada S. Omokawa M. Kadoguchi T. Yokota T.. (2012). Effect of multiple set on intramuscular metabolic stress during low-intensity resistance exercise with blood flow restriction. Eur. J. Appl. Physiol. 112, 3915–3920. doi: 10.1007/s00421-012-2377-x, PMID: 22415101 PMC3474903

[ref88] Takada S. Okita K. Suga T. Omokawa M. Kadoguchi T. Sato T.. (2012). Low-intensity exercise can increase muscle mass and strength proportionally to enhanced metabolic stress under ischemic conditions. J. Appl. Physiol. 113, 199–205. doi: 10.1152/japplphysiol.00149.2012, PMID: 22628373

[ref89] Takano H. Morita T. Iida H. Kato M. Uno K. Hirose K.. (2005). Effects of low-intensity “KAATSU” resistance exercise on hemodynamic and growth hormone responses. Int. J. KAATSU Train. Res. 1, 13–18. doi: 10.3806/ijktr.1.13

[ref90] Takarada Y. Nakamura Y. Aruga S. Onda T. Miyazaki S. Ishii N. (2000a). Rapid increase in plasma growth hormone after low-intensity resistance exercise with vascular occlusion. J. Appl. Physiol. 88, 61–65. doi: 10.1152/jappl.2000.88.1.61, PMID: 10642363

[ref91] Takarada Y. Takazawa H. Ishii N. (2000b). Applications of vascular occlusion diminish disuse atrophy of knee extensor muscles. Med. Sci. Sports Exerc. 32, 2035–2039. doi: 10.1097/00005768-200012000-00011, PMID: 11128848

[ref92] Thiebaud R. S. Yasuda T. Loenneke J. P. Abe T. (2013). Effects of low-intensity concentric and eccentric exercise combined with blood flow restriction on indices of exercise-induced muscle damage. Interv. Med. Appl. Sci. 5, 53–59. doi: 10.1556/IMAS.5.2013.2.1, PMID: 24265891 PMC3831801

[ref93] Vechin F. C. Libardi C. A. Conceição M. S. Damas F. R. Lixandrão M. E. Berton R. P. B.. (2015). Comparisons between low-intensity resistance training with blood flow restriction and high-intensity resistance training on quadriceps muscle mass and strength in elderly. J. Strength Cond. Res. 29, 1071–1076. doi: 10.1519/JSC.0000000000000703, PMID: 25264670

[ref94] Weatherholt A. M. Vanwye W. R. Lohmann J. Owens J. G. (2019). The effect of cuff width for determining limb occlusion pressure: a comparison of blood flow restriction devices. Int. J. Exerc. Sci. 12, 136–143. PMID: 30761200 10.70252/RWVU7100 PMC6355123

[ref95] Wernbom M. Augustsson J. Thomeé R. (2006). Effects of vascular occlusion on muscular endurance in dynamic knee extension exercise at different submaximal loads. J. Strength Cond. Res. 20, 372–377. doi: 10.1519/R-16884.1, PMID: 16686566

[ref96] Wernbom M. Schoenfeld B. J. Paulsen G. Bjørnsen T. Cumming K. T. Aagaard P.. (2020). Commentary: can blood flow restricted exercise cause muscle damage? Commentary on blood flow restriction exercise: considerations of methodology, application, and safety. Front. Physiol. 11:243. doi: 10.3389/fphys.2020.00243, PMID: 32265737 PMC7098946

[ref97] Wilk M. Krzysztofik M. Filip A. Zajac A. Bogdanis G. C. Lockie R. G. (2020). Short-term blood flow restriction increases power output and bar velocity during the bench press. J. Strength Cond. Res. doi: 10.1519/JSC.0000000000003649 [Epub ahead of print]., PMID: 32379236

[ref98] Wilson J. M. Lowery R. P. Joy J. M. Loenneke J. P. Naimo M. A. (2013). Practical blood flow restriction training increases acute determinants of hypertrophy without increasing indices of muscle damage. J. Strength Cond. Res. 27, 3068–3075. doi: 10.1519/JSC.0b013e31828a1ffa, PMID: 23446173

[ref99] Yasuda T. Abe T. Brechue W. F. Iida H. Takano H. Meguro K.. (2010a). Venous blood gas and metabolite response to low-intensity muscle contractions with external limb compression. Metabolism 59, 1510–1519. doi: 10.1016/j.metabol.2010.01.016, PMID: 20199783

[ref100] Yasuda T. Fujita S. Ogasawara R. Sato Y. Abe T. (2010b). Effects of low-intensity bench press training with restricted arm muscle blood flow on chest muscle hypertrophy: a pilot study. Clin. Physiol. Funct. Imaging 30, 338–343. doi: 10.1111/j.1475-097X.2010.00949.x, PMID: 20618358

[ref101] Yasuda T. Loenneke J. P. Ogasawara R. Abe T. (2013). Influence of continuous or intermittent blood flow restriction on muscle activation during low-intensity multiple sets of resistance exercise. Acta Physiol. Hung. 100, 419–426. doi: 10.1556/APhysiol.100.2013.4.6, PMID: 24317348

[ref102] Zhao Y. Lin A. Jiao L. (2020). Eight weeks of resistance training with blood flow restriction improve cardiac function and vascular endothelial function in healthy young Asian males. Int. Health 13, 471–479. doi: 10.1093/inthealth/ihaa089, PMID: 33175117 PMC8417084

